# Supplementary material for: Distinct responses of frond and root to increasing nutrient availability in a floating clonal plant
Source: PLoS One. 2021 Oct 11;16(10):e0258253. doi: 10.1371/journal.pone.0258253 (PMC8504747; doi:10.1371/journal.pone.0258253)
Supplement: Attachment — Submitted filename: Response to Reviewers.docx [file pone.0258253.s002.docx]

**Response to Reviewers**

**Reviewer #1**: It’s an interesting work. The manuscript read fluently. I only have a few small questions.

Response: Thanks.

The authors used 10 levels of Hoagland’s solution. There are some different formulas of Hoagland’s solution. Could the authors show the exact solution composition?

Response: We have added the exact solution composition in the revised version (lines 94 – 98).

Effects of nutrients on plants’ growth have been linked to compositions and concentrations. For *Egeria densa*, ammonia nitrogen blow 1 mg L^-1^ could promote growth of plants, but ammonia nitrogen exceeding 10 mg L-1 might produce oxidative stress to plants and inhibit the growth. For *Vallisneria natans*, the thresholds of promotion and inhibition effects of ammonia nitrogen are respectively lower than 0.6 mg L^-1^ and higher than 1 mg L^-1^. Ammonia nitrogen in high concentrations of Hoagland’s solutions might make an adverse effect on growth, which should be considered and discussed.

Response: Nice point, thanks. We have addressed this issue in the discussion section. (Lines 224 – 227).

There has been study showing that copper exceeding 1 μmol L^-1^ significantly inhibits growth of *Spirodela polyrhiza*. Mn exceeding certain concentration also has negative effect on growth of *Spirodela polyrhiza*. It has been proved by many studies that low concentration of heavy metal could promote growth. Heavy metal elements come from high concentrations of Hoagland’s solutions might also make adverse effects on growth. Some studies have also reported that heavy metal pollution decreased root to shoot ratio. Effects of heavy metal should be considered.

Response: We agree and we have discussed it in the revised version (Lines 224 – 227).

Different elements (ions) have antagonistic or synergistic effects with each other. An antagonistic (synergistic) relationship between elements (ions) at certain concentration may change into a synergistic (antagonistic) relationship at another concentration. For example, Ca^2+^ and K^+^ have antagonistic and competitive effects at normal concentration, while Ca^2+^ can promote the uptake of K^+^ by plants at very low concentration. The relationship between elements (ions) is complex due to composition of Hoagland’s solution, especially at high concentrations. This may have something with the hump-shaped and U-shaped changes.

It is suggested the authors should dive deeper into the experimental phenomenon.

Response: Good to know, thanks. In the revised version, we also discussed the potential relationship between different elements in driving the responses of *Spirodela polyrhiza* to nutrient availability (Lines 227 – 229).

**Reviewer #2**: The manuscript reported a case study that tested the effects of nutrient availability on clonal performance (growth, morphology and biomass allocation) of a floating clonal plant *Spirodela polyrhiza*. The novelty of the paper is to explore plant responses to a wide gradient of nutrient availability (1/64 to 8 × full-strength Hoagland solution), which have rarely been examined in previously published studies. The experimental design is clear, and the content was well edited.

Response: Thanks.

However, when I checked the raw data in the supplementary material, there is one serious problem. It is not logical that the values of root mass and/or root to shoot ratio of *S. polyrhiza* are treated as zero when root length has non-zero values. The reverse situation is also not logical.

If some of data are missing, they should be treated as null values and excluded from the analyses. I suggest that the data should be re-analyzed and re-discussed after they are carefully checked.

Response: We agree and we have reanalyzed our data by excluding the null values, which did not change our results. We have updated the results in the revised version (Lines139 – 145, 165 – 170 and 178 – 181).

Specific comments:

Introduction

Line 17, delete “or”.

Response: Deleted (Line 36).

Materials and methods

Line 49, replace “flower” with “flowers”.

Response: Replaced (Line 69).

Line 69, replace “sizes” with “size”.

Response: Replaced (Line 90).

Line 75, delete “of time”.

Response: Deleted (Line 101).

Line 101, replace “show” with “showed”.

Response: Replaced (Line 131).

Results

Line 111, It appears to be better to use the phrase “4 × full-strength Hoagland solution”, compared to the used one “4-Hoagland solution”, when describing the levels of nutrient availability.

Response: Thanks, we replaced “4-Hoagland solution” with “4 × full-strength Hoagland solution” (Lines 143 – 145). We also changed the phrase throughout the whole manuscript (Lines 6 – 7, 92 – 93, 166 – 170 and 180 – 184).

Discussion

Line 172, use “reasons”, instead of “mechanisms”.

Response: Changed (Line 220).

Line 176 – 178, In your study, the nutrient solutions were refreshed with the four-day interval, so it seems to be sufficient for plant growth during the entire experimental period.

Response: Yes, we agree and we have removed this explanation.

Conclusions

Line 216, delete “a lot”.

Response: Deleted (Line 284).

Line229, “master student”?

Response: Deleted “Master” (Line 272).

References

Line 230 – 438, the reference formats and some spelling errors should be carefully checked and corrected.

For instance, spelling errors: “ence” in line 315; “thr” in 362. Format errors: line 287-288; line 343, line 355-356; line 359; line 364-366; line 367-369; line 378; line 423.

Response: We have checked and reformatted our references in the revised version (Lines 288 –507).
